# Integrin α2-deficient mice provide insights into specific functions of collagen receptors in the kidney

**DOI:** 10.1186/1755-1536-3-19

**Published:** 2010-09-22

**Authors:** Rainer Girgert, Maria Martin, Jenny Kruegel, Nicolai Miosge, Johanna Temme, Beate Eckes, Gerhard-Anton Müller, Oliver Gross

**Affiliations:** 1 Department of Nephrology and Rheumatology, Georg-August-University Goettingen, Goettingen, Germany; 2 Department of Prosthodontics, Tissue Regeneration Work Group, University Medicine, Georg-August-University Goettingen, Goettingen, Germany; 3 Department of Dermatology, University of Cologne, Cologne, Germany

## Abstract

**Background:**

Integrins are important cellular receptors for collagens. Within the glomerulus, podocytes regulate the integrity of the glomerular basement membrane (GBM) by sensing the presence of collagen and regulating collagen IV synthesis. The present study evaluates the role of integrin α2 (ITGA2) in cell-matrix interaction.

**Methods and Results:**

ITGA2-deficient mice had normal renal function but moderate proteinuria and enhanced glomerular and tubulointerstitial matrix deposition. Electron microscopy demonstrated irregular podocyte-matrix interaction, causing pathological protrusions towards the urinary (podocyte) side of the GBM. These characteristic subepithelial bulges mimic the renal phenotype of mice, which are deficient in another collagen receptor, discoidin domain receptor (DDR)1. Using immunogold staining, ITGA2 expression was found to localize to the basolateral site of the podocyte foot processes. ITGA2-deficient mice overexpressed transforming growth factor (TGF)β and connective tissue growth factor (CTGF) compared with wild-type mice. Using *in situ *hybridization, tubular cells were found to be the primary site of TGFβ synthesis and podocytes the source of CTGF in ITGA2-deficient mice.

**Conclusion:**

These findings support our hypothesis that both these collagen receptors (ITGA2 and DDR1) play a similar role within the kidney. Further, cell-matrix interaction via collagen receptors seems to be crucial for maintenance of normal GBM architecture and function. Targeting collagen receptors such as ITGA2 might be a new form of treatment for progressive fibrotic diseases.

## Introduction

Integrins are cellular receptors that are important for sensing the extracellular environment and adjacent cells. Each cell type expresses a characteristic combination of α- and β-subunits. The α-subunit of an integrin predicts its ligand specificity, whereas the cytoplasmic tail of the β-subunit interacts with the cytoskeleton and with proteins involved in signal transduction. Integrin α1 has only one known partner, β1; α1β1 is an important receptor for collagens and laminins in basement membranes [1,2]. The most abundant integrins within the glomerulus are α1β1, α2β1 and α3β1. Integrin α1β1 is expressed in all glomerular cell types [3], and plays a crucial role in cell proliferation and collagen turnover, and in sensing extracellular collagen levels and downregulating endogenous collagen synthesis [4]. α1β1 integrin is expressed primarily on mesangial cells, α2β1 primarily on endothelial cells, and α3β1 on podocytes [5]. Integrin α1-knockout mice are fertile with no overt phenotype [5-7], whereas targeted deletions of α4 and α5 integrins are lethal during embryogenesis [8]. Embryonic fibroblasts derived from α1-deficient animals are unable to spread on collagen IV [9]. The role of α1β1 integrin in collagen-dependent cell proliferation, adhesion, matrix remodeling and mesangial cell migration points to integrins playing a role in the pathogenesis of collagen diseases such as the hereditary type IV collagen disease Alport syndrome (AS).

AS is caused by mutations in the α3/4 or 5 chains of type IV collagen, which result in proteinuria and renal failure [10]. Type IV collagen is the major constituent of the glomerular basement membrane (GBM). During embryogenesis, the premature GBM, consisting of α1/α1/α2 (IV) chains, is produced by endothelial cells and podocytes. The mature GBM, containing α3/α4/α5 (IV) chains, is built solely by podocytes [11-14]. Mutations in the α3/α4/α5 (IV) chains in AS result in characteristic ultrastructural changes in the GBM [10]. One important step in the pathogenesis of AS is thought to be an altered cell-matrix interaction via the podocyte collagen receptors. For example, ASmice, which carry an additional knockout for the integrin α1 gene, show delayed onset and slower disease progression [5].

Integrins can be activated by triple-helix type I and IV collagens [15]. Binding of integrin to its ligands influences the actin cytoskeleton via activation of cdc42, Rac1 and focal adhesion kinase (FAK) [16]. The activation of α1β1 integrin downregulates collagen synthesis [4], suggesting that the functional loss of this integrin could predispose the host to augmented sclerosis after injury. Transforming growth factor (TGF)β is known to stimulate the production of the extracellular matrix (ECM) in podocytes [17]. As integrin-mediated FAK activation leads to the downregulation of TGF-β receptors, loss of α1β1 function results in increased collagen expression [18].

The possible role of collagen receptors other than integrins, such as discoidin domain receptor (DDR)1 in renal disease has been previously addressed by our group [19,20]. Loss of DDR1 delays renal fibrosis in AS [20]. The role of α2β1 integrin in renal pathology is less clear. Examination of integrin α2 knockout mice showed a multifaceted phenotype including defects of branching morphogenesis, hemostasis and a partially defective platelet interaction with collagen [7,21]. The nonlethal phenotype of the α2-deficient mice implies that many of the key functions of α2β1 can be mimicked by other integrins. In the present study, we analyzed the renal phenotype of integrin α2-deficient mice, and found that loss of integrin α2 results in irregular podocyte--matrix interaction, causing pathological protrusions towards the urinary (podocyte) side of the GBM and increased TGFβ and connective tissue growth factor (CTGF) expression.

## Materials and methods

The animal care and experiments were performed according to the Declaration of Helsinki and the Guide for the Care and Use of Laboratory Animals (NIH).

### Knockout mice

The methods for generation of integrin α2 knockout mice and for PCR-based genotyping have been described previously [7,21]. The ITGA2 knockout and wild-type mice (provided by B. Eckes, University of Cologne, Cologne, Germany) used here were bred on a C57Bl6 background under pathogen-free housing conditions at the local animal facility with a 12-h light/dark cycle and unlimited access to food and water. The DDR1^-/- ^mice were provided by W. Vogel, University of Toronto, Toronto, Canada, and the collagen (IV) α3 (COL4A3)^-/- ^mice by Jackson Immunoresearch Laboratories (Westgrove, PA, USA). COL4A3^-/- ^mice were crossbred to the C57Bl6 background for nine generations. No animals were lost due to infections during monitoring. For western blotting, histology and assessment of urea levels, animals were examined at 100 and 150 days of age.

### Western blot analysis

Three to four kidneys from either the wild-type or ITGA2-deficient groups were homogenized in a solution of Tris-buffered saline (TBS) containing a cocktail of protease inhibitors (phenylmethylsulfonyl fluoride (PMSF), leupeptin and pepstatin). Next, 20 μg of protein (equal loading dose shown by the bicinchoninic acid (BCA) protein assay) was solubilized in lithium dodecyl sulfate (LDS) sample buffer and separated in a 4 to 12% gel (NuPage Novex Bis-Tris Gel; Invitrogen, Carlsbad, CA, USA), transferred to a polyvinylidene fluoride membrane, and blocked with 5% dry milk powder in TBS with Triton X-100 (TBST). The membrane was incubated overnight with mouse anti-TGFβ1 (1:2000; R&D Systems, Minneapolis, USA) and rabbit anti-CTGF (1:2000; Abcam Limited, Cambridge, United Kingdom), then with the horseradish peroxidase-conjugated secondary antibody (Dako, Hamburg, Germany) for 1 hour at room temperature. The blots were developed by chemoluminescence using enhanced chemiluminescence (GE-Healthcare, Freiburg, Germany). Protein expression was analyzed by densitometry (Kd1 software; Kodak, Rochester, NY, USA).

### *In situ *hybridization

Paraffin-embedded sections (5 μm thick) were dewaxed and rehydrated in decreasing concentrations of ethanol. After two washes in phosphate-buffered saline (PBS), sections were partially digested with 6 μg/ml proteinase K (Qiagen, Hilden, Germany) for 30 min. Sections were post-fixed with 4% formaldehyde for 5 minutes before hybridization with a digoxigenin (DIG)-labeled RNA probe specific for either TGFβ mRNA or CTGF mRNA. Probes for *in situ *hybridization were labeled with DIG by *in vitro *transcription in the presence of DIG-UTP using reverse transcriptase PCR products of TGFβ and CTGF as templates, linked to promoters of SP6 or T7 RNA polymerase by molecular cloning. DIG-labeled probes were hybridized to the sections in the hybridization solution at 42°C overnight. Subsequently, sections were washed twice in 2 × sodium citrate buffer and PBS. Bound probes were detected with (1:500) rabbit anti-DIG-AP (Roche, Penzberg, Germany) in TBS containing 5% dry milk powder, and visualized using the diluted alkaline phosphatase substrate nitro blue tetrazolium chloride/5-bromo-4-chloro-3'-indolyphosphate p-toluidine salt (NBT/BCIP) (1:50; Roche, Penzberg, Germany) at pH 9.5.

### Immunohistochemistry of tissue sections and scoring of fibrosis

From each group, three to five mice were killed and transcardially perfused with a solution of paraformaldehyde and glutaraldehyde; kidneys were immersion fixed as described previously [8]. Semi-thin and thin sections of paraffin wax-embedded kidney were cut (Ultracut UCT ultramicrotome; Reichert Inc., Depew, NY, USA) into sections 5 μm thick, then dewaxed, rehydrated in graded alcohol and blocked with 5% bovine serum albumin (BSA) in TBS at room temperature. Endogenous peroxidases were blocked by treatment with 3% hydrogen peroxide in PBS for 10 minutes. Primary antibodies (rabbit anti-mouse EHS-laminin and rabbit anti-mouse fibronectin, 1:1000;gift from M. Paulsson, Cologne, Germany) were incubated overnight at 4°C. Next, sections were incubated with goat anti-rabbit Cy3 (Jackson ImmunoResearch Laboratories Inc.) as a secondary antibody and analyzed under a microscope (Axiophot; Zeiss, Göttingen, Germany). Stained sections were scored for protein deposition as described previously [10]. Glomerulosclerosis was defined as the loss of >50% of the glomerular lumen due to matrix accumulation. Tubulointerstitial fibrosis was evaluated in a similar manner by grading by a blinded observer of 12 kidney sections, each from three animals per group, for accumulation of ECM (graded as 0, 1+ and 2+).

### Electron microscopy and immunogold detection of integrin α2

For ultrastructural electron microscopy, kidney samples (4-mm^3^) taken from three mice per group were fixed in Karnovsky solution and embedded in epoxy resin as described previously [22]. Ultrathin sections were prepared (Reichert-Jung Ultracut E; Leica, Wetzlar, Germany) and examined under an electron microscope (LEO 906E; Zeiss, Oberkochen, Germany). For immunogold staining, kidney samples of 1 mm^3 ^were fixed and embedded in a hydrophilic resin (LR-Gold; London Resin Company, Reading, UK) as described previously [23]. Ultrathin sections were blocked with 1% BSA in PBS for 20 min at room temperature. The primary antibody (rabbit anti-mouse integrin α2, 1:50 in PBS; Abcam) was incubated for 1 hour at room temperature. A secondary antibody (goat anti-rabbit IgG, 1:300 in PBS; Medac, Hamburg, Germany) was coupled to 16-nm colloidal gold particles according to standard protocols [24] and incubated for 20 min at room temperature. Contrast staining with 1% uranyl acetate and lead citrate was carried out for 10 min each at room temperature. Negative control experiments were performed using ITGA2^-/- ^kidneys and by treating the sections with 1% BSA instead of the primary antibody. To exclude binding of uncoupled gold particles to tissue structures, control sections were incubated with a pure colloidal gold solution.

### Analyses of urine and serum

Proteins from 20 μl of urine of three to five mice per group were precipitated using methanol/chloroform and dissolved in loading buffer. They were separated in a pre-cast 4 to 12% gradient polyacrylamide gel (Invitrogen) and stained with Coomassie blue, then the protein bands were densitometrically analyzed [19]. The serum urea levels of three t five mice per group was determined using an automatic analyzer (Hitachi 91; Boehringer Mannheim, Germany).

### Statistics

Results are presented S means and standard deviation. Data were analyzed by one-way analysis of variance (ANOVA).

## Results

### Loss of ITGA2 mimics the renal phenotype of DDR1 knockouts (ultrastructural changes in GBM)

**Figure 1:**
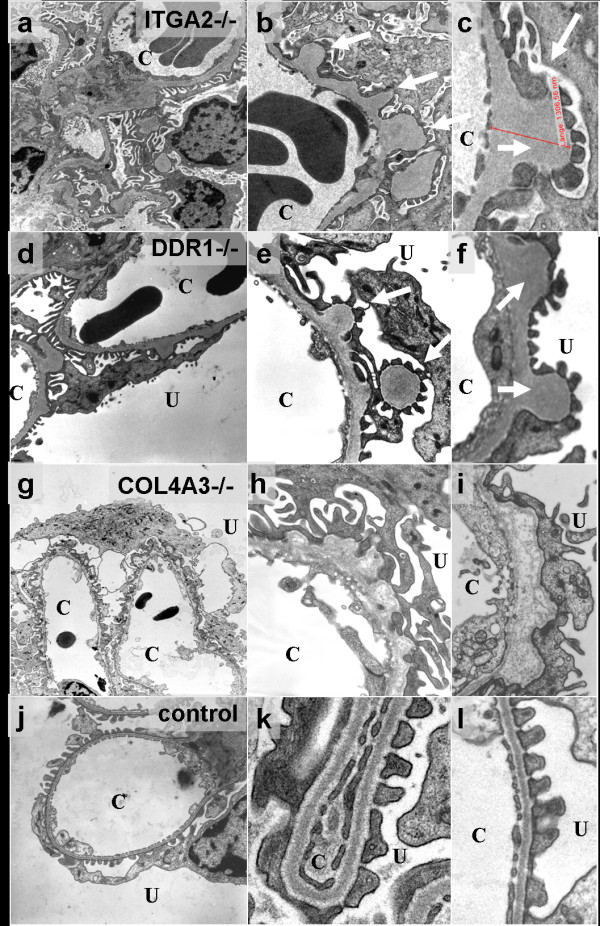
**Ultrastructural analysis of the glomerular basement membrane**. Mice deficient in **(a-c) **integrin α2 (ITGA2), **(d-f) **discoidin domain receptor (DDR)1 and **(g-i) **collagen (IV) α3 (COL4A3), and **(j-l) **wildtype controls. Electron microscopy demonstrates a localized, mushroom-like isodense thickening of the glomerular basement membrane (GBM) in (**a-c**) ITGA2-deficient mice, compared with (**j-l**) wild-type mice. **(b, c) **These characteristic subepithelial bulges (white arrows) seem to be identical to those observed in (**d-f) **DDR1 knockouts [19]) **(g-i) **By contrast, splitting and thickening of the GBM and fibrillar collagens indicating fibrosis were found in COL4A3-deficent mice. Original magnification: **(a, d, g, j) **× 3,000 to 7,000; **(b, c, e, f, h, i, k, l) **× 15,000 to 30,000. U = urinary space; C = capillary lumen.

Electron microscopy demonstrated local GBM thickening (Figure 1b, c, white arrowheads ) in ITGA2 knockouts. These local changes affected 1 to 3% of the total GBM, compared with 100% of the GBM affected in AS (Figure 1g-i) [10]. The protrusions in ITGA2 knockout micestained very homogeneously (Figure 1-c), similar to the protrusions found in DDR1 knockouts (Figure 1d-f) [14]. By contrast, the splitting of the GBM found in AS mice was much more heterogeneous and contained fibrillar collagens (Figure 1h, i). In AS mice, the GBM changes were accompanied by severe loss and effacement of the podocyte foot processes (Figure 1i), whereas the foot processes in ITGA2 knockout mice appeared to be normal and stayed attached to the GBM even in areas of GBM thickening (Figure 1b, c).

### Loss of ITGA2 causes moderate glomerular and tubulointerstitial damage

**Figure 2:**
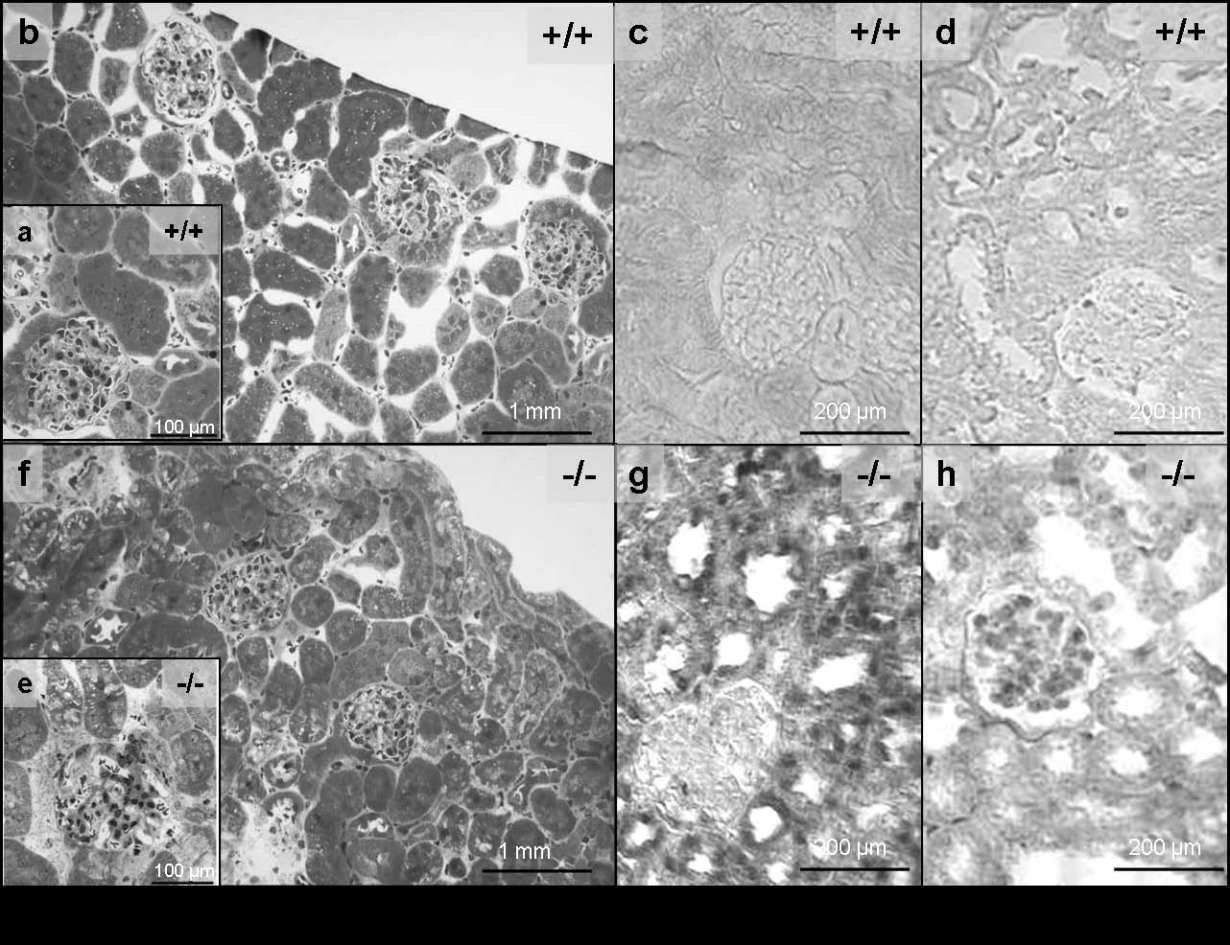
**Renal histology in integrin α2 (ITGA2)-deficient mice**. Compared with **(a, b) **wild-type controls, loss of ITGA2 caused **(e-f) **minor glomerular, periglomerular and tubulointerstitial damage. I*n situ *hybridization localized elevated transforming growth factor (TGF)β expression in ITGA2 knockouts to the tubular space (**g**), whereas (**h**) upregulation of connective tissue growth factor (CTGF) seemed to be focused on the glomerular space.

**Figure 3:**
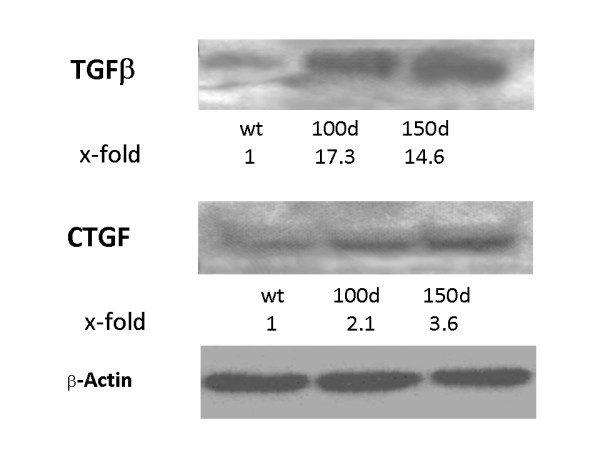
**Upregulation of pro-fibrotic factors**. Integrin α2 (ITGA2) knockouts showed increased levels of transforming growth factor (TGF)β and connective tissue growth factor (CTGF) in whole kidney extracts.

According to our hypothesis, loss of ITGA2 may influence cell-matrix interactions, including profibrotic effects due to the upregulation of profibrotic cytokines. Compared with wild-type controls (Figure 2a, b), 150-day-old ITGA2^-/- ^mice (Figure 1e, f) showed minor glomerular, periglomerular and tubulointerstitial matrix accumulation. In parallel, total kidney extracts from ITGA2^-/- ^mice showed upregulation of TGFβ (Figure 3, lanes 2 and 3), whereas TGFβ in wild-type mice was barely detectable (Figure 3, lane 1). Additionally, the signal for CTGF was increased in ITGA2 knockouts (Figure 2d) compared with wild-type mice. Densitometric evaluation of the TGFβ bands revealed 17.3-fold (100 days) and 14.6-fold (150 days) increases in TGFβ and 2.1-fold and 3.6-fold increases in CTGF in ITGA2 knockout mice (Figure 3).

*In situ *hybridization of TGFβ and CTGF confirmed these results (Figure 2c versus Figure 2g and Figure 2d versus Figure 2h). TGFβ was not detectable in the kidneys of wild-type mice, whereas in ITGA2 knockouts, TGFβ was primarily expressed in tubular cells, with a weak glomerular signal (Figure 2g). Kidneys of wild-type mice were also negative for CTGF, but in ITGA2 knockouts, expression of CTGF was more pronounced in the glomerulus than in tubular cells (Figure 2h).

### Loss of ITGA2 increases glomerular and global tubulointerstitial matrix deposition and fibrosis

**Figure 4:**
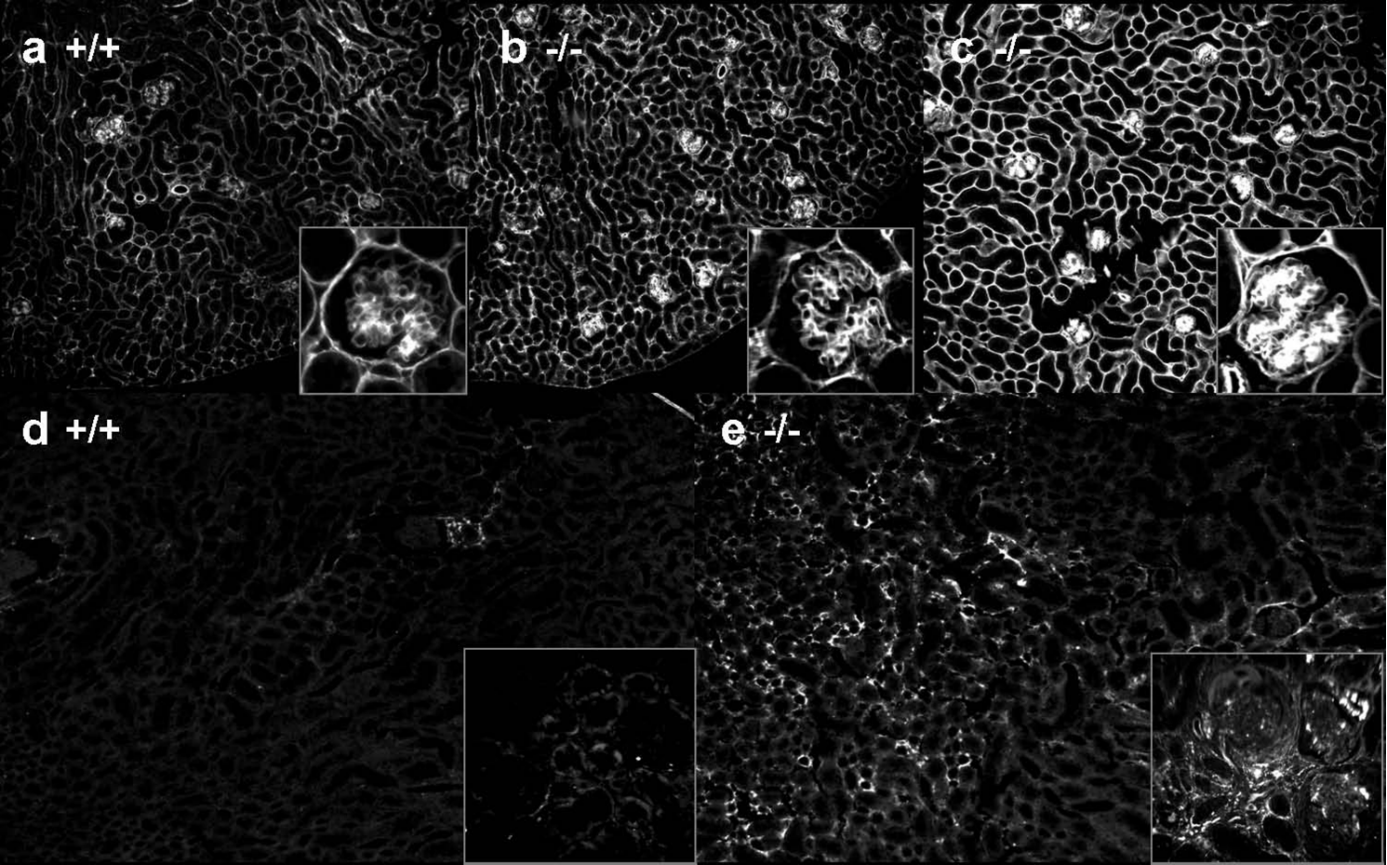
**Immunostaining for extracellular matrix and scar tissue**. **(a-c) **EHS laminin; **(d, e) **fibronectin. Compared with **(a) **150-day-old wild-type control mice, **(b) **100-day-old and **(c) **150-day-old Integrin α2 (ITGA2) knockout mice expressed increased amounts of extracellular matrix. **(e) **In parallel, 150-day-old ITGA2 knockouts showed accumulation of tubulointerstitial and glomerular scar tissue compared with **(d) **wild-type mice of the same age.

To analyze the effects of ITGA2 knockout on the production of ECM and induction of fibrosis, kidney sections of healthy (Figure 4a) and knockout mice (Figure 4b, c) were immunostained for EHS laminin (laminin1), a marker for global glomerular and tubulointerstitial ECM deposition. The glomerular and tubulointerstitial matrix deposition was somewhat increased in 100-day-old ITGA2^-/- ^mice (Figure 4b) compared with wild-type controls (Figure 4a), and this difference was increased further in 150-day-old mice (Figure 4c). Increased laminin-1 synthesis in the GBM of ITGA2^-/- ^mice is shown at higher magnification in Figure 4 (b, c, insets)

Fibronectin staining also demonstrated moderate glomerular, periglomerular and tubulointerstitial deposition in 150-day-old ITGA2^-/- ^mice (Figure 4e) compared with wild-type controls (Figure 4d). The tubulointerstitial fibrosis score (range 0 to 2) was 1.1 at 150 days in ITGA2 knockouts compared with 0.3 in wild-type mice. In parallel, the glomerulosclerosis index (range 0 to 2) increased to 0.7 in ITGA2^-/- ^mice at 150 days compared with 0.4 in their wild-type littermates.

### Localization of integrin α2 expression

**Figure 5:**
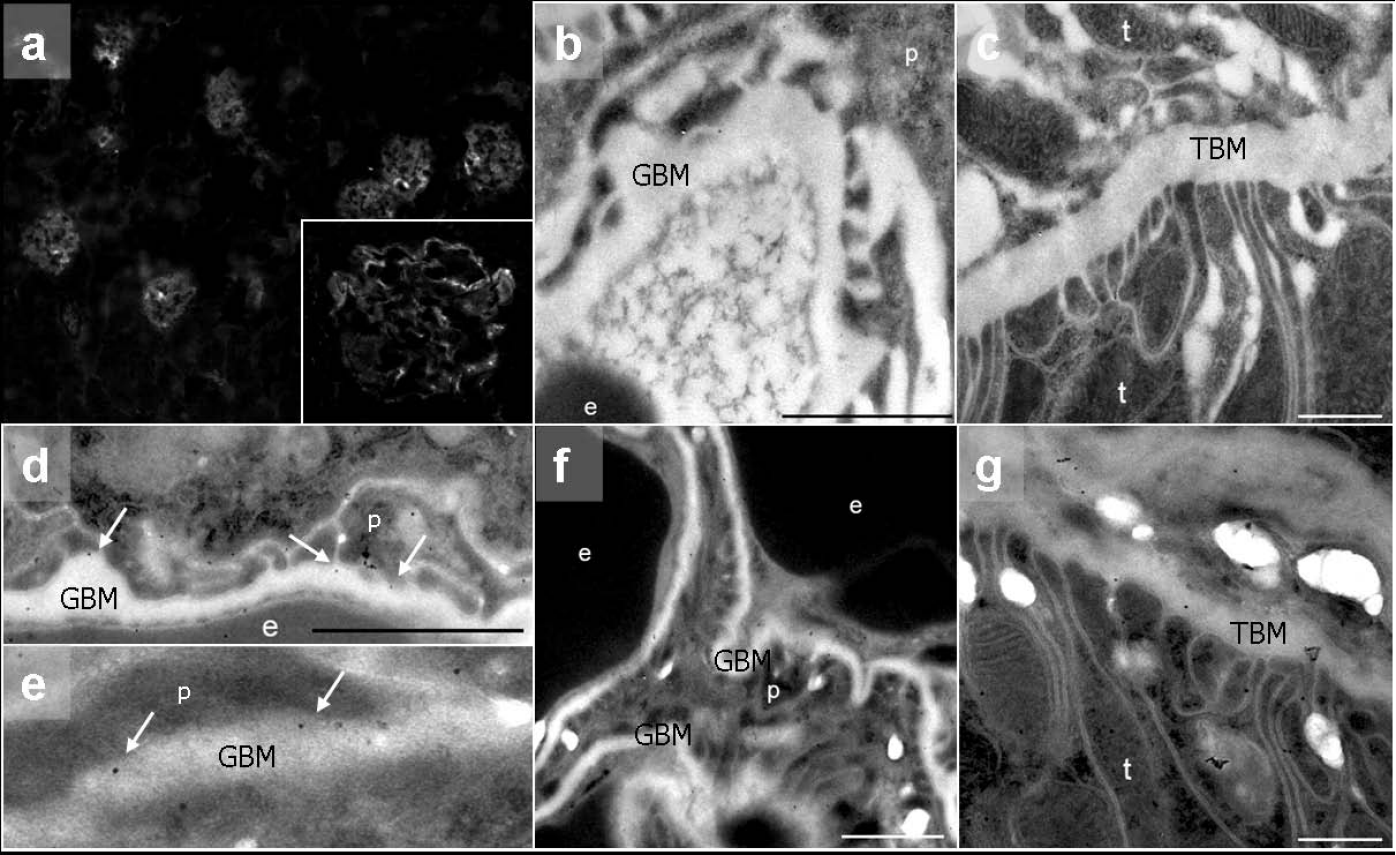
**Localization of integrin α2 (ITGA2) expression**. **(a) **Immunohistochemistry of wild-type mice with an antibody to ITGA2 showed staining solely in the glomeruli, whereas kidney tubules remain unstained. (Inset) At higher magnification, ITGA2 was mainly expressed in cells along the basement membrane, most probably podocytes. **(d, e) **Immunogold localization within the glomerulus demonstrated localization of ITGA2 primarily at the basal side of the podocyte foot processes and close to the glomerular basement membrane (GBM) (white arrows). **(d-f) **ITGA2 was not detected on the surface of endothelial cells. **(b, c) **Negative controls in ITGA2 knockouts. Original magnification: **(a) **× 100, × 400; **(b-g) **20,000 to 30,000. e **= **erythrocyte; p **= **podocyte foot processes; t **= **tubular cell; TBM **= **tubular basement membrane.

Immunostaining localized integrin α2 solely to the glomeruli, whereas the tubules remained unstained (Figure 5a). At higher magnification, cells sitting on the outer side of the GBM, most probably podocytes, expressed integrin α2 (Figure 5a insert). These results were further strengthened by immunogold localization: ITGA2 was found primarily at the basal side of the podocyte foot processes close to the GBM (Figure 5d, e, white arrows ). The surface of podocytes always showed staining for ITGA2, but it was never detected on the surface of endothelial cells (Figure 5d-f).

### GBM ultrastructural changes result in moderate proteinuria

**Figure 6:**
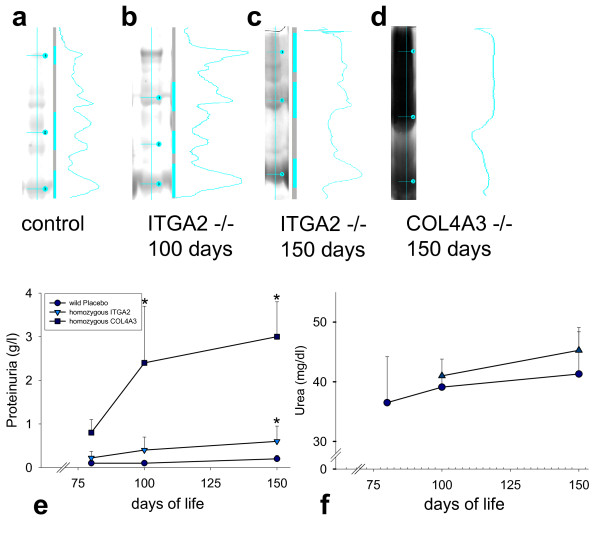
**Proteinuria and renal function in integrin α2 (ITGA2) knockouts**. Electrophoresis of urine proteins reveals middle- and high-molecular weight proteinuria at **(b, c) **100 and 150 days compared with **(a) **healthy wild-type controls. However, the amount of proteinuria in **(e) **ITGA2 knockouts (triangles) wa s much lower than in COL4A3 knockouts (with ongoing renal failure and nephrotic syndrome; squares). **(f) **Levels of serum urea of ITGA2 knockouts (triangles) were not significantly different from those of healthy controls (circles) at 100 and 150 days.

Serum urea level of 150-day-old ITGA2 knockout mice was 45.3 ± 3 mmol/l and was not significantly increased compared with healthy mice (Figure 6f). Further, mutant mice showed moderate proteinuria (Figure 6a-e), with slightly increased excretion of higher molecular weight proteins in the 150-day-old knockout mice (Figure 6c) compared with healthy controls (Figure 6a, which was in contrast to the severe proteinuria of COL4A3^-/- ^mice with ongoing renal failure (Figure 6d).

## Discussion

The present study investigated the role of the collagen receptor integrin α2 in the kidney. Confirming our hypothesis, type IV collagen receptors were able to downregulate *de novo *collagen synthesis as long as the GBM was intact. This hypothesis was strengthened by the renal phenotype of DDR1-knockout mice (Figure 1d-f) [19]. We were able to demonstrate that the loss of integrin α2 expression induces localized matrix overproduction in the GBM similarly to DDR1 knockouts. These changes lead to moderate proteinuria but are not sufficiently severe to cause further renal damage and eventual renal failure. Interestingly, the loss of ITGA2 mimics the renal phenotype of DDR1 knockouts, indicating a similar role of both collagen receptors within the kidney.

In patients with mutated type IV collagen genes, receptors become unable to recognize collagen correctly. As a consequence, signals suppressing collagen synthesis are depleted, inducing non-physiological overproduction (with faulty collagen chains such as α1/α2 type IV collagen chains, fibrillar collagens, and various laminins) [12]. In chronic renal fibrotic diseases such as AS, glomerular and tubulointerstitial matrix deposition and scarring are regulated by TGFβ and CTGF. The present study shows that loss of the collagen receptor integrin α2 increases the total amounts of renal TGFβ and CTGF. TGFβ is known to stimulate the production of ECM in podocytes [25], thus inducing GBM thickening in the ITGA2 knockouts. These alternations of the GBM lead to proteinuria, which in turn eads to secondary tubular damage and tubulointerstitial fibrosis. However, it remains unclear whether this proteinuria triggers upregulation of TGFβ and other chemokines or if the global loss of ITGA2 itself leads to tubulointerstitial fibrosis. Future studies should concentrate on the role of ITGA2 in renal disease and on the possible balancing role of both ITGA2 and DDR1.

Within the glomerulus, bioactive TGF-β1 is derived from the serum of the capillary lumen or from glomerular cells [22]. TGFβ upregulates type IV collagen in all glomerular cell types [25], and increases promoter activity of fibronectin and α2 type I collagen genes. Further, a consensus TGF-β element is located in the promoter of the rat pro α1 type I collagen gene [26]. TGF-β induces the expression of CTGF via a functional Smad3 binding site in the CTGF promoter. In diabetic nephropathy, CTGF has been reported to play an important role in glomerular alteration by inducing the production of fibronectin and of collagen types I and IV [27].

According to our hypothesis, podocytes do not only produce mature GBM components (α3/4/5(IV) collagen) [13], but also react to altered GBM composition via collagen receptors, leading to matrix overproduction. Integrin α1β1 is crucial for downregulating collagen synthesis, suggesting that its functional loss could enhance renal fibrosis [28]. Additionally, in the present study, the localization of integrin α2 at the lateral base of podocytes points to an important role of integrin α2 in the cell-matrix interaction between the GBM and podocytes. ITGA2 seems not to be crucial for embryogenesis. However, cell-matrix interaction via tyrosine kinase receptors such as DDR1 and ITGA2 seems to be very important to the mature kidney, otherwise different collagen receptors compensating each other's role would not exist. Future experiments should focus on the balanced function and interaction of DDR1 and ITGA2.

Abrahamson *et al. *recently demonstrated the distinct role of the podocyte in the preservation and production of mature GBM components [13]. This group further verified that collagen receptors, particularly integrins, are linked to the cytoskeleton of podocytes. The activation of DDR1 has been shown to influence smooth muscle cell migration and matrix metalloproteinase expression [29]; both mechanisms play an important role in the pathogenesis of AS [5,10,12,30,31]. Future experiments should focus on the role of collagen receptors such as integrin α2 in the architecture and cytoskeleton of podocytes. Further, interaction between the GBM, podocytes and slit membrane might become increasingly important in the pathogenesis of chronic fibrotic renal diseases and the pathogenesis (and therapy) of proteinuria.

Our data provide evidence that altered cell-matrix interaction via collagen receptors such as α2 integrin and DDR1 are one important step in the maintenance and production of ECM and in the sequence of fibrogenesis. Thus, targeting collagen receptors might be important in the treatment of progressive fibrotic diseases.

## Conflict of interests statement

The authors declare that they have no competing interests.

## Authors' contributions

The manuscript was drafted by RG and OG; the study was conceived by MM, JK, NM and JT; the study's design and coordination was done by RG, NMi, BE, G-AM and OG. All authors read and approved the final manuscript.
